# Calibrated, explainable machine learning on routine laboratory data to characterize diagnostic assignment patterns in rheumatic diseases: a retrospective study of 12,085 patients

**DOI:** 10.1186/s41927-025-00607-7

**Published:** 2025-12-29

**Authors:** Amal Mohamed Elmesiry, Amira Shahin Ibrahim, Hemmat A. Elabd, Basma Mohamed El Naggar, Eman E. Abd Elsalam, Mai Abd El Halim Moussa, Eman A. Rageh, Mona Mokhtar, Muhammad M. Harb, Aya H. Elshazly, Mohamed A. Khalafallah, Atef A. Hassan

**Affiliations:** 1 Department of Rheumatology and Rehabilitation, Faculty of Medicine for Girls, Al Azhar University, Cairo, Egypt; 2 Department of Rheumatology and Rehabilitation, Faculty of Medicine, Al Azhar University, Cairo, Egypt; 3 Department of Internal Medicine, Faculty of Medicine for Girls, Al-Azhar University, Cairo, Egypt; 4 Faculty of Medicine, Alexandria University, Alexandria, Egypt; 5 Faculty of Medicine, Al-Azhar University, Cairo, Egypt

**Keywords:** Rheumatology, Differential diagnosis, Machine learning, Explainable AI, SHAP, Calibration, Multiclass classification, Spondyloarthritis, Seronegative, Laboratory biomarkers

## Abstract

**Background:**

Overlap in routine laboratory profiles complicates differential diagnosis of rheumatic diseases, particularly seronegative spondyloarthritis. We examined whether models trained on routine labs reproduce diagnostic assignment patterns and yield calibrated, explainable probabilities.

**Methods:**

We analyzed a publicly available, fully de-identified dataset (*n* = 12,085). Adults (≥ 18 years) with confirmed diagnoses and ≤ 30% biomarker missingness were included. Nineteen routine variables (demographics, ESR/CRP, serology) plus four engineered features were used. Missingness (~ 14.5%) was imputed using MICE, variables were standardized, and the data were split 80/20 with stratification. Random Forest, LightGBM, XGBoost, CatBoost, and TabNet were trained with fixed, literature-informed hyperparameters. We assessed 5-fold CV, independent test performance, calibration (Brier/ECE), and SHAP; a predefined seronegative subgroup (RF/anti-CCP negative) was analyzed.

**Results:**

XGBoost achieved the highest test accuracy (85.48%); Random Forest (83.78%) was selected for detailed interpretation due to superior calibration. Performance varied by disease: SLE recall was 97.9% compared to ankylosing spondylitis (AS), 57.6%. Among 2,417 test cases, 381 (15.76%) were misclassified; the most frequent error was AS misclassified as RA (109; 28.6%). SHAP ranked ESR/CRP, RF/anti-CCP, HLA-B27, and C3/C4 as dominant contributors. In seronegative patients (*n* = 390), the prevalence of HLA-B27 was higher (+ 6.5%; *p* = 0.024), and the prevalence of anti-La was lower (–11.6%; *p* = 0.001).

**Conclusions:**

Routine laboratory data can be converted into calibrated, explainable probabilities that characterize diagnostic assignment patterns, rather than independent predictions. Given poor AS performance, the approach is not reliable for differentiating spondyloarthropathies from RA without additional clinical or imaging data. External/temporal validation, integration of clinical and imaging features, and prospective evaluation are needed.

**Trial registration:**

Not applicable.

**Supplementary Information:**

The online version contains supplementary material available at 10.1186/s41927-025-00607-7.

## Introduction

Differentiating common rheumatic diseases remains challenging because clinical phenotypes and routine biomarkers often overlap, resulting in diagnostic delays and misclassification at the point of care, especially within the spondyloarthritis spectrum, where imaging and longitudinal context are crucial. Recent syntheses highlight both the persistence of diagnostic delays in axial spondyloarthritis and the parallel risks of over- or misdiagnosis when imaging is scarce or inconsistently interpreted, underscoring the need for scalable data-driven support tools [1].

Across rheumatology, machine learning (ML) and broader AI methods are increasingly utilized for classification, risk stratification, and treatment guidance, leveraging data from tabular laboratories, imaging, and multi-omics. However, concerns about generalizability, calibration, and transparency remain central barriers to its clinical adoption [2–4]. In parallel, reporting guidance has evolved. TRIPOD + AI emphasizes explicit handling of missingness, internal and external validation, probability calibration, and decision-analytic evaluation to support safe clinical translation [5].

Importantly, contemporary work demonstrates that models trained only on readily available laboratory indicators can distinguish early systemic autoimmune rheumatic diseases with promising discrimination, suggesting a practical path for settings in which advanced imaging or specialty testing is not immediately available [6]. Explainable AI methods such as SHAP further enable biomarker-level accountability by linking predictions to clinically recognizable drivers, thereby addressing a key adoption hurdle in high-stakes diagnostics [7].

Building on these advances, we analyzed a large, fully de-identified repository of 12,085 individuals with routine biomarker profiles and confirmed diagnoses made openly available for reproducible ML benchmarking via Harvard Dataverse (dataset record, mirrored listing, and accompanying description) [8].

Because many of the laboratory variables used here directly inform clinical diagnosis (e.g., RF/anti-CCP for RA, complements for SLE, HLA-B27 for axial SpA), our analysis is susceptible to incorporation (circularity) bias: features lie on the causal pathway from disease to diagnostic labeling. Accordingly, we frame our aim as characterizing how routinely collected biomarkers align with diagnostic assignment and producing calibrated probabilities that could support differential diagnosis, rather than claiming independent prediction divorced from clinical practice. Prospective, pre-diagnostic studies are needed to evaluate the independent predictive utility.

Our objective was to develop and validate modern tabular ML models using only routinely collected demographics and serologies, quantify discrimination and calibration, interrogate decision logic with explainability methods, and probe seronegative presentations where conventional serology underperforms, thereby assessing whether accessible lab data can be translated into transparent, calibrated probabilities that support earlier, more accurate differential diagnoses across seven rheumatic conditions.

## Methods

### Study design and data source

This study was a retrospective diagnostic analysis designed to develop and validate machine learning models for the differential diagnosis of seven rheumatic diseases. We used a publicly available anonymized dataset sourced from the Harvard Dataverse repository [8]. The dataset contained 12,085 de-identified patient records, each with a corresponding biomarker profile and a confirmed diagnosis.

### Ethical considerations

All analyses used a publicly available, fully de-identified dataset from Harvard Dataverse, and complied with the dataset’s stated license and terms of use, as posted on the repository record [8]. No attempt has been made to reidentify individuals or link records to external data sources. The results are reported only in aggregate, and potentially identifying small cell sizes was avoided or suppressed where relevant.

### Study population and variables

The inclusion criteria stipulated a confirmed diagnosis of one of the seven rheumatic diseases or a healthy control status, an age of ≥ 18 years, and a biomarker profile with ≤ 30% missing data.

Nineteen predictors were used, including demographic variables (age and sex), inflammatory markers (ESR and CRP), and a panel of 11 serological markers (RF, Anti-CCP, HLA-B27, ANA, Complement C3, Complement C4). Four features were clinically engineered, including the Inflammation Score (ESR + CRP level). The target variable for prediction was the disease diagnosis, which was treated as a 7-class categorical outcome.

### Data handling and preprocessing

Missing data were a significant consideration, affecting approximately 14.5% of all data points, primarily in specialized serological tests, such as anti-Sm (43.0% missing). A detailed breakdown of missingness by variable is provided in Supplementary Table S1. We assumed that the data were Missing At Random (MAR) because test ordering is typically guided by clinical suspicion. All missing values were imputed using Multivariate Imputation by Chained Equations (MICE) [9]. Following imputation, continuous variables were standardized (standard scale). The cohort was split into training (*n* = 9,668; 80%) and test (*n* = 2,417; 20%) sets using stratified random sampling [10].

### Model development and validation

Five supervised machine learning models were created: Random Forest [11], LightGBM [12], XGBoost [13], CatBoost [14], and TabNet [15]. Hyperparameters were set in advance (not adjusted on the test set) to regularize the models and restrict their capacity—for example, Random Forest (n_estimators = 500, max_depth = 8, min_samples_leaf = 20), XGBoost (eta = 0.05, max_depth = 5, subsample = 0.8, colsample_bytree = 0.8), and LightGBM (num_leaves = 31, feature_fraction = 0.8, bagging_fraction = 0.8).

Model performance was assessed using an independent test set. Internal validation was confirmed using 5-fold stratified cross-validation, and the detailed fold-by-fold results demonstrating model stability are shown in Supplementary Table S2. Statistical comparisons between the models were performed using McNemar’s test [16]. Explainability analysis was conducted using SHAP (Shapley Additive exPlanations) to identify the clinical drivers of model predictions. Subgroup analysis was performed on seronegative patients (RF- and anti-CCP-negative) to identify alternative diagnostic pathways [17].

### Software and reproducibility

All analyses were conducted using Python 3.12, with a random state of 42 to ensure reproducibility. The computational performance, including the training time and model size, for each of the five models is reported in Supplementary Table S3. This report adhered to the TRIPOD + AI guidelines [5].

## Results

### Patient population

The study cohort included 12,085 patients with 9,668 (80%) in the training set and 2,417 (20%) in the test set. The cohort included 2,848 (23.6%) patients with Rheumatoid Arthritis (RA) and 2,123 (17.6%) with Ankylosing Spondylitis (AS). A clinically significant subgroup of 390 (3.2%) seronegative patients was identified in this study. The complete demographic and disease distributions of the cohort are presented in Table 1.

**Table 1:** Study population characteristics

Characteristic	Total Cohort (*n* = 12,085)
Age, years	49.91 ± 17.65
Gender, n (%)	
Female	5,934 (49.1%)
Male	6,151 (50.9%)
Disease Distribution, n (%)	
Rheumatoid Arthritis	2,848 (23.6%)
Ankylosing Spondylitis	2,123 (17.6%)
Sjögren’s Syndrome	1,850 (15.3%)
Psoriatic Arthritis	1,784 (14.8%)
Normal/Controls	1,614 (13.4%)
Systemic Lupus Erythematosus	1,353 (11.2%)
Reactive Arthritis	513 (4.2%)
Seronegative Patients	390 (3.2%)
Data Split	
Training set	9,668 (80.0%)
Test set	2,417 (20.0%)

### Overall model classification performance

All five models showed high classification accuracy on the independent test set, as indicated in Table 2. The XGBoost model achieved the highest accuracy (85.48%), with no signs of overfitting (+ 0.32% gap). The Random Forest model was chosen for the next detailed analysis due to its solid balance of accuracy (83.78%) and calibration.

**Table 2:** Overall model performance comparison

Model	Training Accuracy	CV Accuracy (± SD)	Test Accuracy	Overfitting Gap	Rank
XGBoost	**85.16%**	**83.94% (± 0.92%)**	**85.48%**	**-0.32%**	1
TabNet	84.47%	82.89% (± 0.86%)	84.44%	+ 0.03%	2
Random Forest	84.82%	82.17% (± 0.58%)	83.78%	+ 1.03%	3
LightGBM	86.92%	82.30% (± 0.78%)	82.79%	+ 4.13%	4
CatBoost	79.41%	79.34% (± 0.56%)	79.85%	-0.44%	5

### Statistical validation and model reliability

A statistical comparison using McNemar’s test revealed no significant difference in classification error rates among the top three models (Random Forest, XGBoost, and LightGBM; all *p* > 0.05), as detailed in Supplementary Table S4. All models showed excellent discriminative ability for most diseases, with macro-averaged Area Under the Curve (AUC) scores exceeding 0.92 (Supplementary Table S5).

**Fig. 1:**
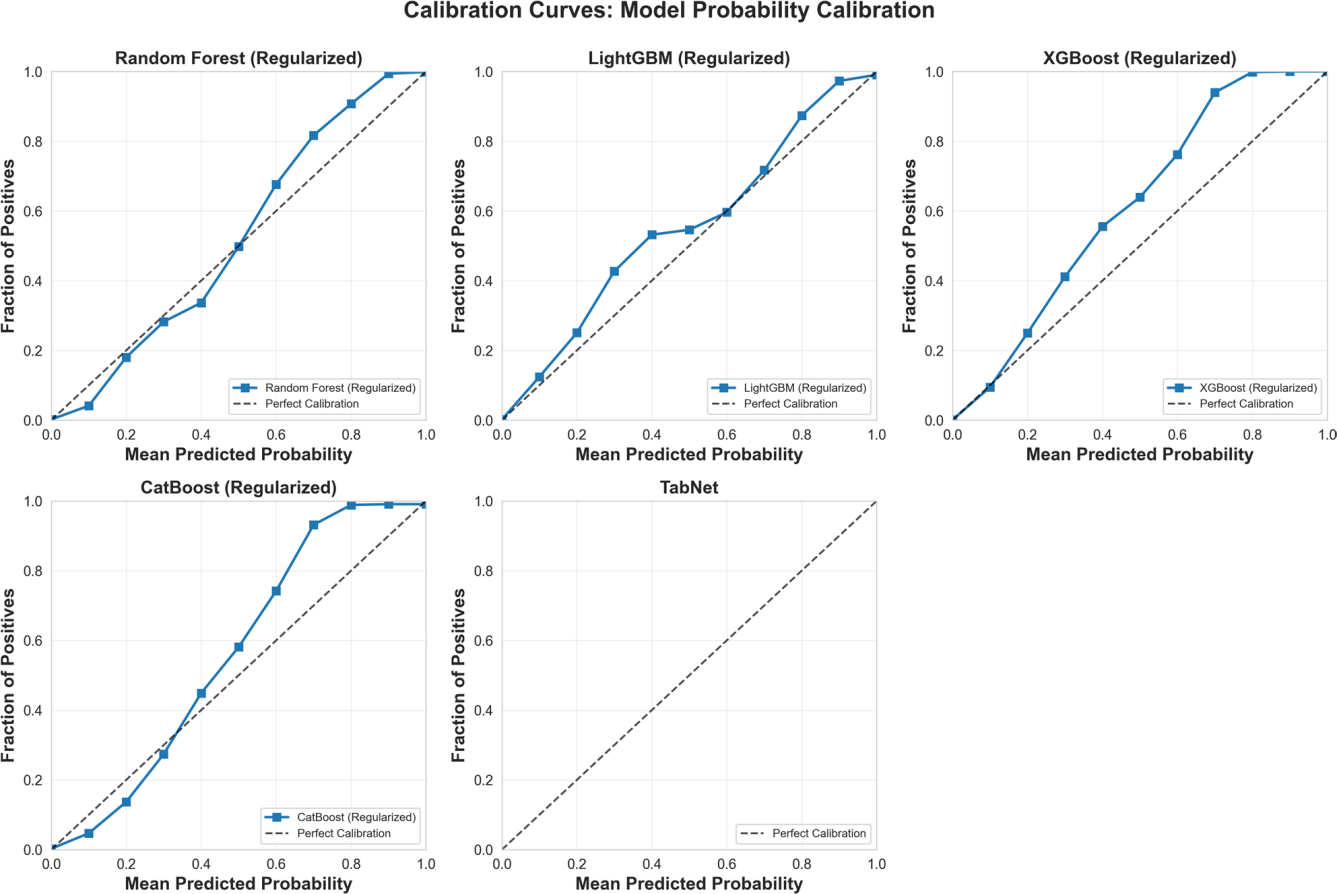
Model Calibration Curves. Description: Shows the calibration plots for all 5 models, comparing mean predicted probability to the actual fraction of positives to assess the reliability of probability scores

Model calibration was assessed for clinical utility. The calibration curves in Fig. 1 demonstrate a close alignment between the predicted probabilities and actual patient outcomes. This visual finding was supported by excellent quantitative metrics, including low Brier scores and Expected Calibration Errors (ECE) for all models (Supplementary Table S6).

### Clinical drivers of diagnosis (explainability)

**Fig. 2:**
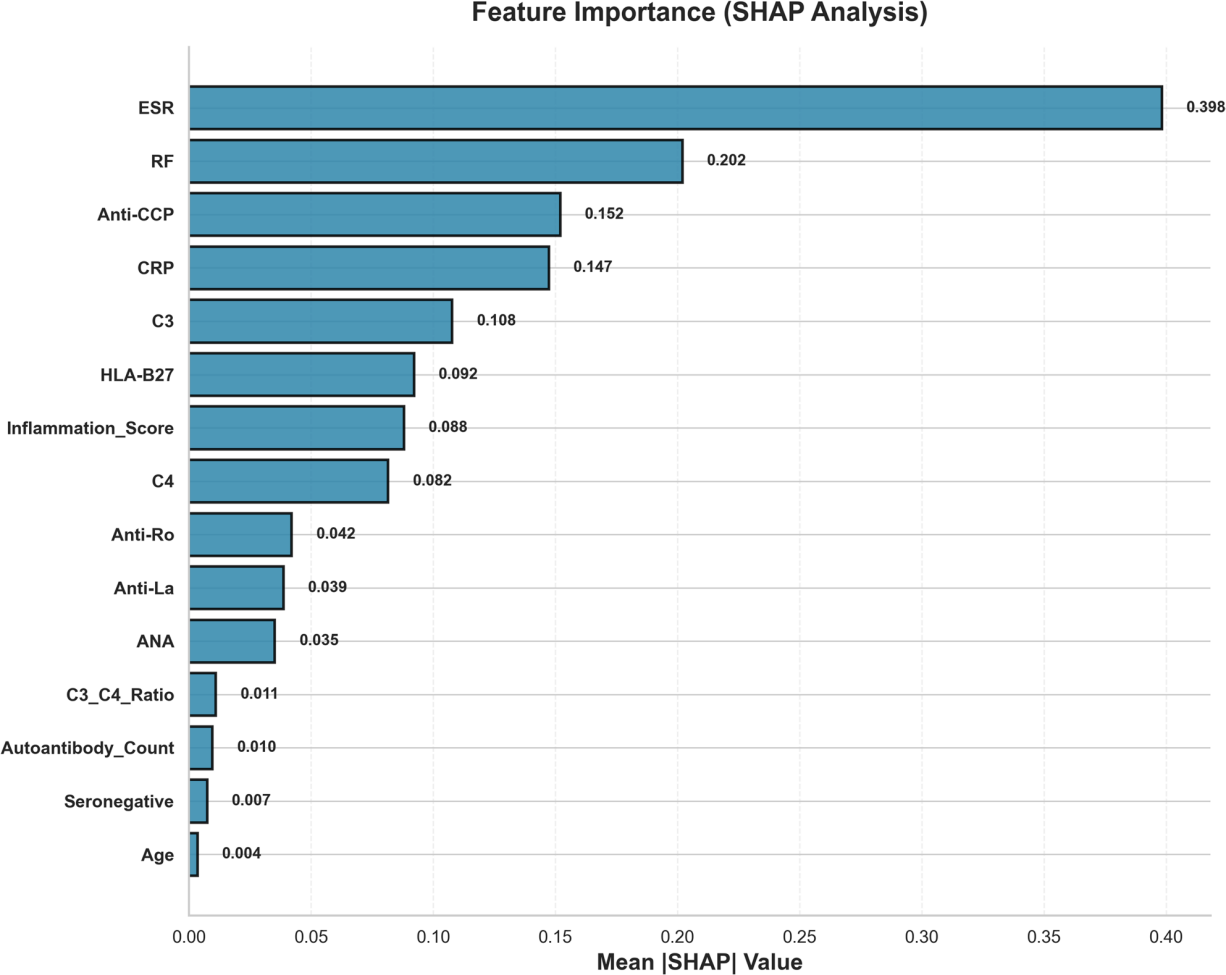
Feature Importance (SHAP Analysis). Description: A bar chart ranking the top clinical and laboratory features by their mean absolute SHAP value, indicating their overall importance in driving the models’ predictions

Explainability analysis identified key biomarkers that drive the model’s diagnostic logic. Figure 2 provides a visual ranking of these features, showing nonspecific inflammatory markers (ESR and CRP) and key autoantibodies (RF and anti-CCP) as the most dominant factors.

Table 3 provides the complete list, confirming that the model’s logic aligns with the established clinical pathophysiology: ESR (mean SHAP 0.398) was the most critical feature, followed by RF (0.202) and anti-CCP (0.152) for RA, and HLA-B27 (0.092) for spondyloarthropathies. Complement proteins C3 (0.108) and C4 (0.082) played a key role in identifying SLE. A comprehensive breakdown of the SHAP values for all 19 features is presented in Supplementary Table S7.

**Table 3:** Feature importance (SHAP Values)

Rank	Feature	Mean |SHAP| Value	Relative Importance	Clinical Interpretation
1	ESR	0.398	100%	Non-specific inflammation marker; elevated in most rheumatic diseases
2	RF	0.202	51%	RA-specific; highly predictive of RA vs. other conditions
3	Anti-CCP	0.152	38%	RA-specific; >95% specificity for RA
4	CRP	0.147	37%	Acute phase reactant; complements ESR
5	C3	0.108	27%	Complement consumption in SLE/autoimmune disease
6	HLA-B27	0.092	23%	Spondyloarthropathy marker (AS, PsA, Reactive)
7	Inflammation Score	0.088	22%	Combined ESR + CRP; overall inflammation burden
8	C4	0.082	21%	Complement; often low in SLE
9	Anti-Ro	0.042	11%	Sjögren’s and SLE marker
10	Anti-La	0.039	10%	Sjögren’s-specific (higher specificity than Anti-Ro)
11	ANA	0.035	9%	Non-specific; present in multiple autoimmune diseases
12	C3/C4 Ratio	0.011	3%	SLE marker (low ratio when both consumed)
13	Autoantibody Count	0.010	2%	Severity/polyautoimmunity indicator
14	Seronegative	0.007	2%	Identifies seronegative arthropathy group
15	Age	0.004	1%	Minimal importance: diseases span age ranges
16	Anti-Sm	0.002	< 1%	SLE-specific but rare; low impact
17	Anti-dsDNA	0.001	< 1%	SLE-specific but low prevalence
18	Gender	0.0004	< 1%	Minimal importance; models disease-agnostic to sex
19	Classical_RA_Positive	0.000	0%	Redundant with RF + Anti-CCP individual values

### Per-class classification performance

The Classification performance of the Random Forest model varied significantly across the seven diseases, reflecting the known clinical challenges (Table 4). The model was exceptionally effective in identifying Systemic Lupus Erythematosus (SLE), achieving 100.0% precision and 97.9% recall (sensitivity). The most significant diagnostic challenge was Ankylosing Spondylitis (AS), which had the lowest recall (57.6%), indicating that 42.4% of patients with AS were misclassified. A comprehensive comparison of the per-class metrics for all five models is presented in Supplementary Table S8.

**Table 4:** Per-class performance metrics (Random Forest, test set *n* = 2,417)

Disease	Precision	Recall	F1-Score	Specificity	Sensitivity	PPV	NPV	Support
SLE	**100.0%**	**97.9%**	**98.9%**	**100.0%**	**97.9%**	**100.0%**	**99.7%**	271
Sjögren’s	84.7%	91.1%	87.8%	97.0%	91.1%	84.7%	98.4%	370
PsA	84.7%	90.2%	87.4%	97.2%	90.2%	84.7%	98.3%	357
Normal	87.7%	81.9%	84.7%	98.2%	81.9%	87.7%	97.3%	321
RA	81.5%	91.4%	86.2%	93.6%	91.4%	81.5%	97.2%	570
Reactive	71.6%	80.6%	75.8%	98.6%	80.6%	71.6%	99.1%	103
AS	**76.8%**	**57.6%**	**65.9%**	**96.3%**	**57.6%**	**76.8%**	**91.4%**	425
Mean	83.9%	84.4%	83.8%	97.3%	84.4%	83.9%	97.3%	-
SD	± 7.4%	± 11.3%	± 9.2%	± 2.0%	± 11.3%	± 7.4%	± 2.7%	-

### Analysis of classification errors

**Fig. 3:**
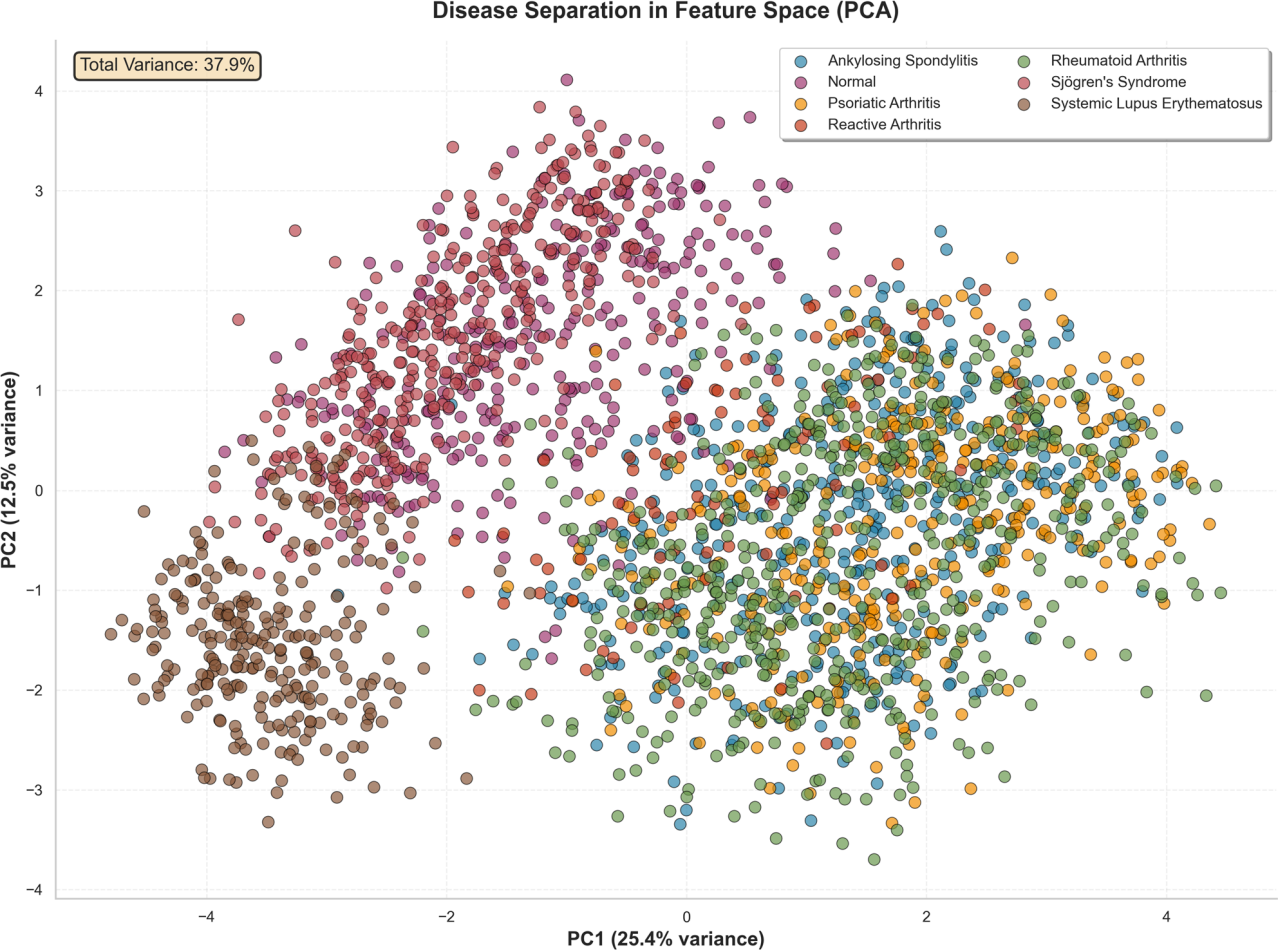
Disease Separation in Feature Space (PCA). Description: A Principal Component Analysis (PCA) plot visualizing all 7 disease classes, illustrating the inherent biomarker overlap between certain diseases (like AS, PsA, and Reactive Arthritis) and the distinct separation of others (like SLE)

**Fig. 4:**
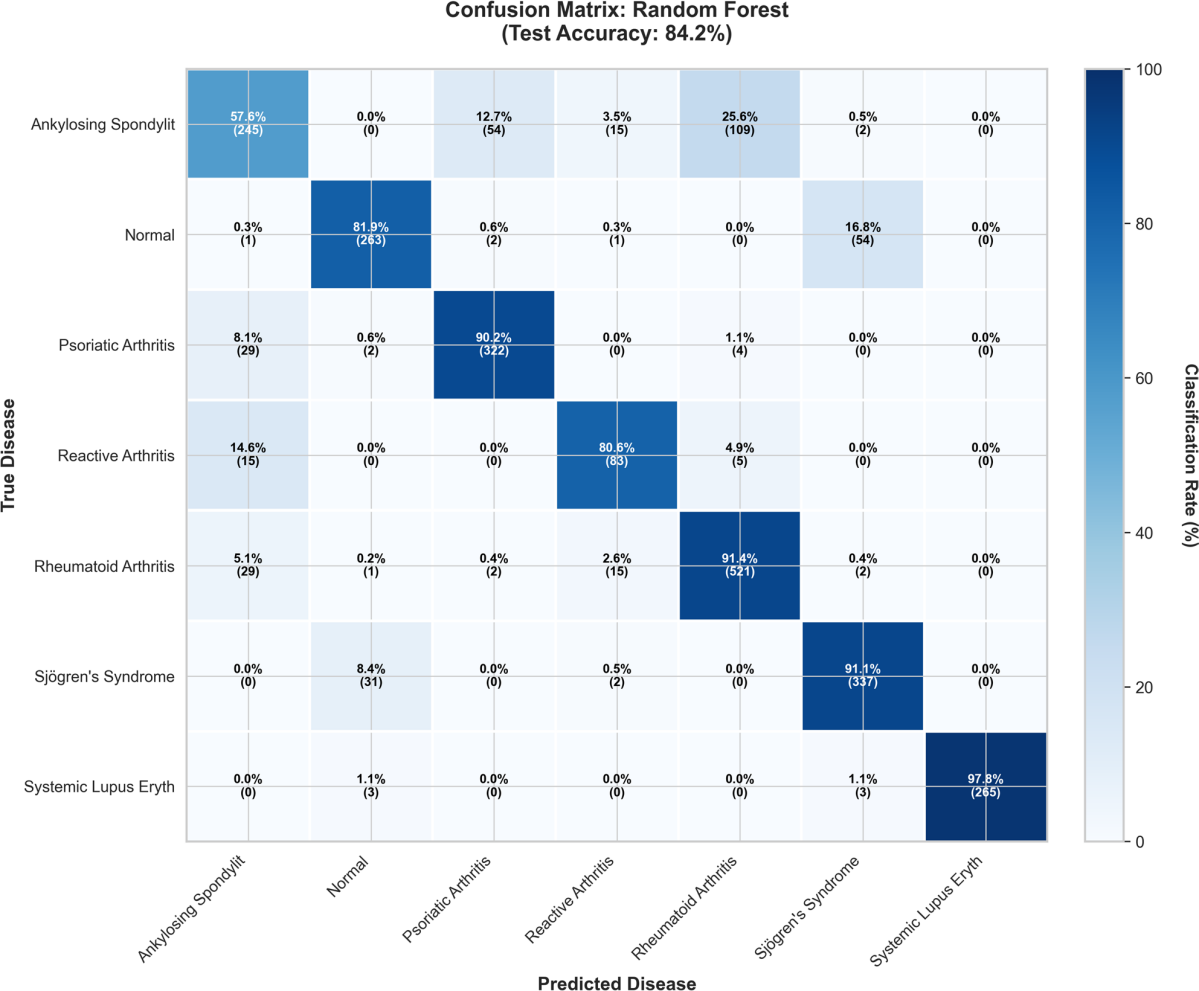
Confusion Matrix (Random Forest). Description: A detailed 7 × 7 confusion matrix for the selected Random Forest model (Test Accuracy: 83.78%), showing the classification rate (percentage) and raw count (number) of patients for all predictions

The Principal Component Analysis (PCA) plot (Fig. 3) shows the natural separation of the classes: SLE forms a compact, well-separated group, while the seronegative spondyloarthropathies (AS, PsA, and reactive arthritis) occupy an overlapping area of feature space. This overlap causes most classification errors. Out of 2,417 test cases, 381 (15.76%) were misclassified. The confusion matrix (Fig. 4) and ranked error pairs (Table 5; Supplementary Figure S1) indicate that the most common error was predicting AS as RA (109 cases; 28.6% of all errors). These patterns align with the model explanations (Fig. 2), where inflammation markers and RF/anti-CCP have a more substantial influence than HLA-B27, resulting in a higher likelihood of assigning cases to RA. Full cell counts for the confusion matrix are available in Supplementary Table S9.

**Table 5:** Top 10 misclassification patterns

Rank	True Disease	Predicted Disease	Errors (*n*)	% of All Errors	% of True Class	Clinical Explanation
1	**AS**	**RA**	**109**	**28.6%**	**25.6%**	Overlapping inflammatory profiles; seronegative RA mimics AS
2	AS	PsA	54	14.2%	12.7%	Both HLA-B27 + spondyloarthropathies
3	Normal	Sjögren’s	54	14.2%	16.8%	Early/subclinical Sjögren’s with low-titer antibodies
4	Sjögren’s	Normal	31	8.1%	8.4%	Mild presentation, borderline serology
5	PsA	AS	29	7.6%	8.1%	Shared spondyloarthropathy features
6	RA	AS	29	7.6%	5.1%	Seronegative RA with axial involvement
7	RA	Reactive	15	3.9%	2.6%	Similar inflammatory pattern
8	Reactive	AS	15	3.9%	14.6%	HLA-B27 association
9	AS	Reactive	15	3.9%	3.5%	Both seronegative spondyloarthropathies
10	Reactive	RA	5	1.3%	4.9%	Post-infectious inflammatory arthritis
**Top 10 Total**			**356**	**93.4%**	-	-

### Insights from the seronegative patient subgroup

Analysis of 390 seronegative patients revealed critical insights into this hard-to-diagnose population.

**Fig. 5:**
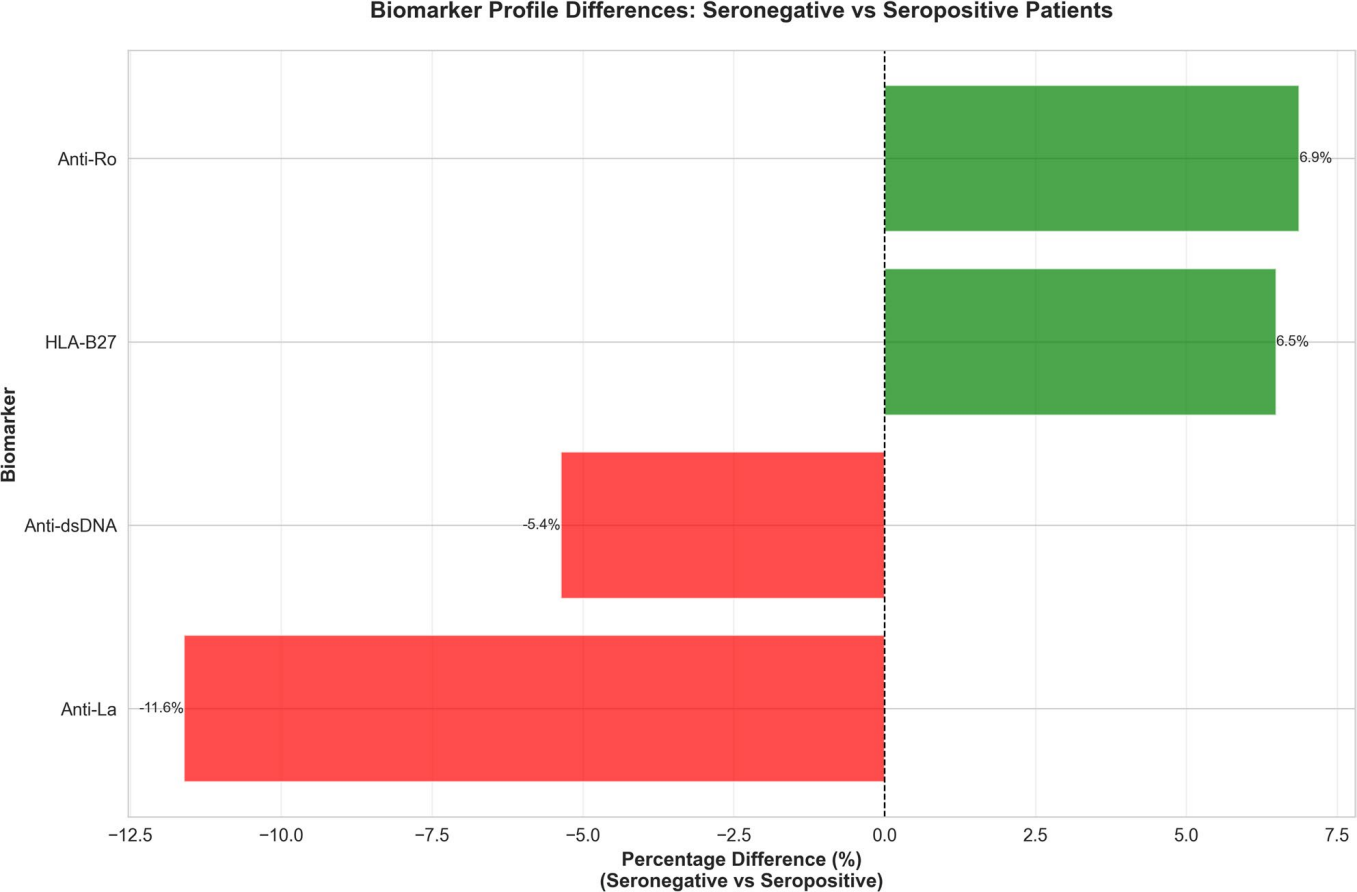
Biomarker Profile Differences (Seronegative vs. Seropositive). Description: A bar chart showing the statistically significant percentage difference in prevalence for key biomarkers (e.g., Anti-La, HLA-B27) when comparing the seronegative subgroup to the main seropositive cohort

- **Biomarker Differences**: As shown in Fig. 5, seronegative patients had a 6.5% higher prevalence of **HLA-B27** (*p* = 0.024) and an 11.6% lower prevalence of **anti-La** (*p* = 0.001) compared to the seropositive group. Full comparison data, which confirmed no significant differences in inflammatory markers, are shown in Supplementary Table S10.

- **Seronegative Subtypes**: This subgroup is not uniform. Clustering revealed four distinct clinical subtypes (Supplementary Figure S2). Detailed biomarker profiles for these clusters are provided in Supplementary Table S11, corresponding to (1) a “High HLA-B27/High Inflammation” group (likely AS), (2) an “Anti-Ro+” group (likely Sjögren’s), (3) a “Low-Marker” group (likely seronegative RA or Normal), and (4) an “ANA+/Low Complement” group (likely SLE or Undifferentiated CTD).
- **Alternative Diagnostic Markers**: A separate SHAP analysis of this subgroup confirmed that in the absence of RF and anti-CCP, **HLA-B27** becomes the single most important diagnostic marker, followed by ESR, CRP, and ANA.

## Discussion

This **descriptive diagnostic** analysis demonstrates that tabular machine-learning models trained on routine laboratory data recover recognizable diagnostic assignment patterns and produce calibrated probabilities that may support multi-disease differential diagnosis across common rheumatic conditions, while maintaining good discrimination and calibration in an independent test set. These observations are clinically plausible: acute-phase reactants (ESR and CRP) and RA-related autoantibodies (RF and anti-CCP) are central to the 2010 ACR/ 2019 EULAR RA classification framework, and SLE classification is associated with high weight to ANA entry plus complement consumption, whereas HLA-B27 is a defining genetic marker in axial spondyloarthritis classification schemes [18, 19].

The explainability profile is also consistent with clinical knowledge: SHAP theory provides locally faithful, consistent attributions for tree-based models, which helps align model logic with established disease biology when inflammatory markers and autoantibodies dominate the importance rankings [17].

A recurring challenge was the diagnostic separation within the spondyloarthritis spectrum, particularly for presumed seronegative presentations where biomarker overlap is expected and classification criteria rely on HLA-B27 and imaging patterns rather than serum autoantibodies.

Finally, the models’ favorable calibration is clinically meaningful because probability estimates can inform threshold-based decisions (e.g., “test-and-refer” strategies), a requirement emphasized by the reporting guidance for prediction models in clinical AI [5, 20].

Our study extends the growing body of literature that uses readily available laboratory variables to stratify systemic autoimmune/rheumatic diseases. A recent multicenter study in *Lupus Science & Medicine* classified early-stage systemic autoimmune rheumatic diseases using accessible laboratory indicators and reported intense discrimination, supporting the feasibility of lab-only models for early differential diagnosis [21].

Relative to this work, the present analysis leverages a larger, fully de-identified public dataset from Harvard Dataverse spanning multiple rheumatic conditions and evaluates several modern tabular learners (gradient-boosting variants, random forests, and a neural tabular model) while pairing performance with global and local explainability grounded in SHAP theory [17].

Our findings are also consistent with disease-specific paradigms: RA models tend to weigh RF/anti-CCP and acute-phase reactants, SLE models weigh ANA/low complements, and spondyloarthritis models benefit most from HLA-B27 patterns that mirror contemporary classification frameworks rather than relying on niche biomarkers [22].

Taken together, these results support the pragmatic role of lab-based ML as an adjunct to clinician judgment, particularly for triage and early diagnostic guidance when definitive findings (e.g., imaging changes and characteristic exam features) are pending. This is directionally aligned with guideline-based constructs: RA classification leverages serology and acute-phase reactants; SLE incorporates ANA entry and complement; and axial SpA elevates HLA-B27. This suggests that such models may operationalize existing clinical knowledge into calibrated probabilities for shared decision-making.

### Strengths and limitations

This study has several strengths that enhance its credibility and potential clinical relevance, as it leverages a large, fully de-identified, publicly available dataset that spans multiple rheumatic conditions, enabling a pragmatic, multiclass differential diagnosis setting. The modeling strategy emphasizes reproducibility (pre-specified hyperparameters, independent test evaluation, and internal cross-validation) and clinical interpretability via SHAP, which provides locally faithful and consistent attributions for tree-based learners. In addition, we assessed probability calibration alongside discrimination and followed contemporary reporting guidance for AI-enabled prediction models, aligning model reporting with best practices intended to support clinical translation.

Significant limitations of this study should also be acknowledged. First, the analysis relied on a single curated public repository. However, large and diverse, a one-source design may not capture cross-institutional differences in test utilization, disease prevalence, or care pathways.

A central limitation is the underperformance in ankylosing spondylitis (recall 57.6%), as well as broader mixing among spondyloarthropathies, with AS→RA being the most common error (28.6%). This is plausibly driven by (i) the dominance of RF/anti-CCP and inflammation markers in the global model logic, which favors RA, and (ii) modest HLA-B27 signal and the absence of imaging/clinical patterning that typically disambiguates axial SpA. Consequently, the current model should not be used to differentiate spondyloarthropathies from RA without additional data.

Second, although we performed internal validation, we did not conduct temporal or geographic external validation, which is specifically recommended for ensuring the trustworthy deployment and transportability of our results.

Third, missing data were handled under a Missing-At-Random (MAR) assumption using multivariate imputation by chained equations. Violations of MAR (e.g., clinically informative test-ordering patterns tied to unobserved factors) could bias imputations, motivating sensitivity analyses to alternative mechanisms.

Fourth, our feature set was restricted to demographics and laboratory biomarkers. In contrast, contemporary classification frameworks for RA, SLE, and axial spondyloarthritis integrate clinical features and, for spondyloarthritis, imaging. Therefore, excluding these modalities may particularly hinder the recognition of seronegative spondyloarthropathy phenotypes. Relatedly, we observed error concentration in spondyloarthritis, which is biologically plausible given the shared inflammatory signatures and the centrality of HLA-B27 and imaging in discriminating against axial disease; incorporating imaging, structured symptom trajectories, and physical examination findings may mitigate this limitation.

Fifth, Incorporation (circularity) bias is an additional limitation: many predictors (RF/anti-CCP, complements, HLA-B27) are on the causal pathway to diagnostic labeling. Therefore, our models mainly identify correlates of the diagnostic process rather than proving independent predictive validity. Prospective, pre-diagnostic evaluation and external/temporal validation are necessary to confirm transportability and independent prediction.

Sixth, pairwise model comparisons used McNemar’s test on dichotomized outcomes. While standard for paired classification error, this does not contrast complete probability distributions or clinical utility across thresholds, supporting the complementary use of decision curve analysis and net benefit reporting in future work.

Seventh, we did not pre-specify or evaluate fairness metrics or calibration across demographic strata; given known age and sex effects on autoimmunity, stratified performance and recalibration should accompany external validation.

Finally, as a retrospective diagnostic study, we did not measure prospective clinical impact; future prospective evaluations should quantify the effects on diagnostic delay, downstream testing, treatment initiation, and model-in-the-loop behaviors with appropriate safety monitoring.

### Future directions

External, multi-site validation with temporal holdouts; additive data modalities (clinical features, imaging), particularly for suspected seronegative spondyloarthritis; prospectively defined decision thresholds with decision-curve analysis; fairness auditing and recalibration by subgroup; and pre-registered implementation studies are warranted to confirm transportability and clinical utility.

## Conclusion

Using a large, de-identified dataset, we developed and validated several machine-learning models that demonstrated intense discrimination and well-calibrated probabilities for distinguishing between the seven rheumatic diseases. Model explanations aligned with known pathophysiology and error patterns, especially within the spondyloarthritis spectrum, and seronegative presentations were clinically plausible. These findings support lab-based ML as a supplemental tool that identifies diagnostic patterns and provides calibrated probabilities; however, additional clinical and imaging data, along with external validation, are needed before relying on this approach to differentiate spondyloarthropathies from RA. The next steps are clear: rigorous external and temporal validation, integration of clinical and imaging data to resolve ambiguity in spondyloarthritis, prospective evaluation of workflow impact and safety, and predefined fairness assessments with recalibration across demographic groups.

## Supplementary Information

Below is the link to the electronic supplementary material.

Supplementary Material 1

Supplementary Material 2

Supplementary Material 3

Supplementary Material 4

Supplementary Material 5

Supplementary Material 6

Supplementary Material 7

Supplementary Material 8

Supplementary Material 9

Supplementary Material 10

Supplementary Material 11

Supplementary Material 12

Supplementary Material 13

## Data Availability

The source dataset is openly available at the Harvard Dataverse (10.7910/DVN/VM4OR3). The analysis code, exact package versions, and environment files used to reproduce all results will be made publicly available when needed. Additional materials (e.g., TRIPOD+AI checklist, supplementary tables/figures) are provided as supplementary files.

## Declarations

### Reporting guidelines

This study adheres to the TRIPOD + AI recommendations for reporting prediction models that use machine learning.
