# Supplementary material for: Calibrated, explainable machine learning on routine laboratory data to characterize diagnostic assignment patterns in rheumatic diseases: a retrospective study of 12,085 patients
Source: BMC Rheumatol. 2025 Dec 29;10:10. doi: 10.1186/s41927-025-00607-7 (PMC12849087; doi:10.1186/s41927-025-00607-7)
Supplement: Supplementary file 1 — Supplementary Material 1 [file 41927_2025_607_MOESM1_ESM.docx]

**Supplementary Table S2: Cross-Validation Performance Details**

| Model | Fold 1 | Fold 2 | Fold 3 | Fold 4 | Fold 5 | Mean | SD | Coefficient of Variation |
| --- | --- | --- | --- | --- | --- | --- | --- | --- |
| XGBoost | 84.2% | 83.5% | 85.1% | 82.8% | 84.3% | 83.94% | 0.92% | 1.10% |
| TabNet | 83.6% | 82.1% | 84.2% | 81.8% | 82.8% | 82.89% | 0.86% | 1.04% |
| Random Forest | 82.8% | 81.5% | 83.1% | 81.2% | 82.3% | 82.17% | 0.58% | 0.71% |
| LightGBM | 83.1% | 81.8% | 84.2% | 80.9% | 81.5% | 82.30% | 0.78% | 0.95% |
| CatBoost | 80.1% | 79.2% | 80.5% | 78.8% | 79.1% | 79.34% | 0.56% | 0.71% |
