# Supplementary material for: Calibrated, explainable machine learning on routine laboratory data to characterize diagnostic assignment patterns in rheumatic diseases: a retrospective study of 12,085 patients
Source: BMC Rheumatol. 2025 Dec 29;10:10. doi: 10.1186/s41927-025-00607-7 (PMC12849087; doi:10.1186/s41927-025-00607-7)
Supplement: Supplementary file 2 — Supplementary Material 2 [file 41927_2025_607_MOESM2_ESM.docx]

**Supplementary Table S3: Computational Performance**

| Model | Training Time | Prediction Time (2,417 samples) | Memory Usage | Model Size |
| --- | --- | --- | --- | --- |
| Random Forest | 42.3 sec | 0.18 sec | 245 MB | 156 MB |
| LightGBM | 18.7 sec | 0.09 sec | 128 MB | 42 MB |
| XGBoost | 35.2 sec | 0.14 sec | 198 MB | 86 MB |
| CatBoost | 52.8 sec | 0.21 sec | 312 MB | 125 MB |
| TabNet | 284.5 sec | 1.42 sec | 892 MB | 248 MB |
