# Supplementary material for: Calibrated, explainable machine learning on routine laboratory data to characterize diagnostic assignment patterns in rheumatic diseases: a retrospective study of 12,085 patients
Source: BMC Rheumatol. 2025 Dec 29;10:10. doi: 10.1186/s41927-025-00607-7 (PMC12849087; doi:10.1186/s41927-025-00607-7)
Supplement: Supplementary file 3 — Supplementary Material 3 [file 41927_2025_607_MOESM3_ESM.docx]

**Supplementary Table S1: Missing Data Pattern**

| Variable | Total Missing | % Missing | Missing in Training | Missing in Test | Imputation Method |
| --- | --- | --- | --- | --- | --- |
| Anti-Sm | 5,197 | 43.0% | 4,158 | 1,039 | MICE (Logistic) |
| Anti-dsDNA | 4,713 | 39.0% | 3,770 | 943 | MICE (Logistic) |
| ANA | 3,746 | 31.0% | 2,997 | 749 | MICE (Logistic) |
| Anti-CCP | 3,263 | 27.0% | 2,610 | 653 | MICE (Logistic) |
| Anti-La | 3,021 | 25.0% | 2,417 | 604 | MICE (Logistic) |
| Anti-Ro | 2,900 | 24.0% | 2,320 | 580 | MICE (Logistic) |
| C3 | 1,692 | 14.0% | 1,354 | 338 | MICE (Bayesian Ridge) |
| C4 | 2,054 | 17.0% | 1,643 | 411 | MICE (Bayesian Ridge) |
| HLA-B27 | 1,934 | 16.0% | 1,547 | 387 | MICE (Logistic) |
| RF | 1,329 | 11.0% | 1,063 | 266 | MICE (Logistic) |
| ESR | 892 | 7.4% | 714 | 178 | MICE (Bayesian Ridge) |
| CRP | 748 | 6.2% | 598 | 150 | MICE (Bayesian Ridge) |
| Age | 0 | 0% | 0 | 0 | None |
| Gender | 0 | 0% | 0 | 0 | None |
| Engineered | 0 | 0% | 0 | 0 | Calculated post-imputation |
