# Supplementary material for: Calibrated, explainable machine learning on routine laboratory data to characterize diagnostic assignment patterns in rheumatic diseases: a retrospective study of 12,085 patients
Source: BMC Rheumatol. 2025 Dec 29;10:10. doi: 10.1186/s41927-025-00607-7 (PMC12849087; doi:10.1186/s41927-025-00607-7)
Supplement: Supplementary file 4 — Supplementary Material 4 [file 41927_2025_607_MOESM4_ESM.docx]

**Supplementary Table S4: McNemar's Test - Pairwise Model Comparisons**

| Model 1 | Model 2 | Model 1 Acc | Model 2 Acc | P-value | Significant | Interpretation |
| --- | --- | --- | --- | --- | --- | --- |
| Random Forest | LightGBM | 84.24% | 83.74% | 0.399 | No | Statistically equivalent |
| Random Forest | XGBoost | 84.24% | 83.24% | 0.076 | No | Statistically equivalent |
| Random Forest | **CatBoost** | **84.24%** | **15.96%** | **<0.001** | **Yes*** | **RF significantly better** |
| LightGBM | XGBoost | 83.74% | 83.24% | 0.363 | No | Statistically equivalent |
| LightGBM | **CatBoost** | **83.74%** | **15.96%** | **<0.001** | **Yes*** | **LightGBM significantly better** |
| XGBoost | **CatBoost** | **83.24%** | **15.96%** | **<0.001** | **Yes*** | **XGBoost significantly better** |
