# Supplementary material for: Calibrated, explainable machine learning on routine laboratory data to characterize diagnostic assignment patterns in rheumatic diseases: a retrospective study of 12,085 patients
Source: BMC Rheumatol. 2025 Dec 29;10:10. doi: 10.1186/s41927-025-00607-7 (PMC12849087; doi:10.1186/s41927-025-00607-7)
Supplement: Supplementary file 5 — Supplementary Material 5 [file 41927_2025_607_MOESM5_ESM.docx]

**Supplementary Table S5: ROC-AUC Scores (Macro-Averaged)**

| Model | Overall AUC | AS | Normal | PsA | Reactive | RA | Sjögren's | SLE |
| --- | --- | --- | --- | --- | --- | --- | --- | --- |
| XGBoost | 0.952 | 0.881 | 0.965 | 0.967 | 0.942 | 0.972 | 0.978 | 0.998 |
| Random Forest | 0.948 | 0.872 | 0.958 | 0.964 | 0.938 | 0.968 | 0.975 | 0.995 |
| LightGBM | 0.945 | 0.865 | 0.952 | 0.961 | 0.935 | 0.965 | 0.972 | 0.993 |
| TabNet | 0.941 | 0.858 | 0.948 | 0.957 | 0.928 | 0.961 | 0.968 | 0.991 |
| CatBoost | 0.928 | 0.842 | 0.935 | 0.948 | 0.915 | 0.952 | 0.961 | 0.989 |
