# Supplementary material for: Calibrated, explainable machine learning on routine laboratory data to characterize diagnostic assignment patterns in rheumatic diseases: a retrospective study of 12,085 patients
Source: BMC Rheumatol. 2025 Dec 29;10:10. doi: 10.1186/s41927-025-00607-7 (PMC12849087; doi:10.1186/s41927-025-00607-7)
Supplement: Supplementary file 6 — Supplementary Material 6 [file 41927_2025_607_MOESM6_ESM.docx]

**Supplementary Table S6: Model Calibration Metrics**

| Model | Brier Score | ECE | Calibration Quality |
| --- | --- | --- | --- |
| Random Forest | **0.0289** | **0.0320** | **Excellent** |
| XGBoost | 0.0297 | 0.0456 | Excellent |
| CatBoost | 0.0305 | **0.0258** | Excellent (Best ECE) |
| LightGBM | 0.0324 | 0.0689 | Good |
