# Supplementary material for: Calibrated, explainable machine learning on routine laboratory data to characterize diagnostic assignment patterns in rheumatic diseases: a retrospective study of 12,085 patients
Source: BMC Rheumatol. 2025 Dec 29;10:10. doi: 10.1186/s41927-025-00607-7 (PMC12849087; doi:10.1186/s41927-025-00607-7)
Supplement: Supplementary file 7 — Supplementary Material 7 [file 41927_2025_607_MOESM7_ESM.docx]

**Supplementary Table S7: Complete Feature Importance Rankings (All 19 Features)**

| Rank | Feature | Mean \|SHAP\| | Variance | Importance Category | Disease Association |
| --- | --- | --- | --- | --- | --- |
| 1 | ESR | 0.398 | 0.042 | Critical | All rheumatic diseases |
| 2 | RF | 0.202 | 0.038 | Critical | Rheumatoid Arthritis |
| 3 | Anti-CCP | 0.152 | 0.031 | Critical | Rheumatoid Arthritis |
| 4 | CRP | 0.147 | 0.029 | High | All inflammatory diseases |
| 5 | C3 | 0.108 | 0.024 | High | SLE, autoimmune |
| 6 | HLA-B27 | 0.092 | 0.022 | High | Spondyloarthropathies |
| 7 | Inflammation Score | 0.088 | 0.019 | High | Overall inflammation |
| 8 | C4 | 0.082 | 0.018 | High | SLE, autoimmune |
| 9 | Anti-Ro | 0.042 | 0.012 | Moderate | Sjögren's, SLE |
| 10 | Anti-La | 0.039 | 0.011 | Moderate | Sjögren's |
| 11 | ANA | 0.035 | 0.010 | Moderate | Multiple autoimmune |
| 12 | C3/C4 Ratio | 0.011 | 0.004 | Low | SLE |
| 13 | Autoantibody Count | 0.010 | 0.003 | Low | Polyautoimmunity |
| 14 | Seronegative | 0.007 | 0.002 | Low | Seronegative arthropathies |
| 15 | Age | 0.004 | 0.001 | Minimal | Non-specific |
| 16 | Anti-Sm | 0.002 | 0.001 | Minimal | SLE (specific but rare) |
| 17 | Anti-dsDNA | 0.001 | 0.000 | Minimal | SLE (specific but rare) |
| 18 | Gender | 0.000 | 0.000 | Minimal | Non-discriminative |
| 19 | Classical_RA_Positive | 0.000 | 0.000 | Redundant | Derived feature |
