# Supplementary material for: Calibrated, explainable machine learning on routine laboratory data to characterize diagnostic assignment patterns in rheumatic diseases: a retrospective study of 12,085 patients
Source: BMC Rheumatol. 2025 Dec 29;10:10. doi: 10.1186/s41927-025-00607-7 (PMC12849087; doi:10.1186/s41927-025-00607-7)
Supplement: Supplementary file 8 — Supplementary Material 8 [file 41927_2025_607_MOESM8_ESM.docx]

**Supplementary Table S8: Detailed Per-Class Metrics for All Models**

| **Model** | **Disease** | **Precision** | **Recall** | **F1-Score** | **Specificity** | **Support** |
| --- | --- | --- | --- | --- | --- | --- |
| **Random Forest** |  |  |  |  |  |  |
|  | Ankylosing Spondylitis | 0.768 | 0.576 | 0.659 | 0.963 | 425 |
|  | Normal | 0.877 | 0.819 | 0.847 | 0.982 | 321 |
|  | Psoriatic Arthritis | 0.847 | 0.902 | 0.874 | 0.972 | 357 |
|  | Reactive Arthritis | 0.716 | 0.806 | 0.758 | 0.986 | 103 |
|  | Rheumatoid Arthritis | 0.815 | 0.914 | 0.862 | 0.936 | 570 |
|  | Sjögren's Syndrome | 0.847 | 0.911 | 0.878 | 0.970 | 370 |
|  | Systemic Lupus Erythematosus | 1.000 | 0.978 | 0.989 | 1.000 | 271 |
| **LightGBM** |  |  |  |  |  |  |
|  | Ankylosing Spondylitis | 0.706 | 0.628 | 0.665 | 0.944 | 425 |
|  | Normal | 0.850 | 0.850 | 0.850 | 0.977 | 321 |
|  | Psoriatic Arthritis | 0.854 | 0.885 | 0.869 | 0.974 | 357 |
|  | Reactive Arthritis | 0.750 | 0.757 | 0.754 | 0.989 | 103 |
|  | Rheumatoid Arthritis | 0.825 | 0.874 | 0.848 | 0.943 | 570 |
|  | Sjögren's Syndrome | 0.872 | 0.884 | 0.878 | 0.977 | 370 |
|  | Systemic Lupus Erythematosus | 1.000 | 0.978 | 0.989 | 1.000 | 271 |
| **XGBoost** |  |  |  |  |  |  |
|  | Ankylosing Spondylitis | 0.723 | 0.588 | 0.649 | 0.952 | 425 |
|  | Normal | 0.867 | 0.832 | 0.849 | 0.980 | 321 |
|  | Psoriatic Arthritis | 0.842 | 0.868 | 0.855 | 0.972 | 357 |
|  | Reactive Arthritis | 0.761 | 0.680 | 0.718 | 0.990 | 103 |
|  | Rheumatoid Arthritis | 0.797 | 0.907 | 0.848 | 0.929 | 570 |
|  | Sjögren's Syndrome | 0.858 | 0.900 | 0.879 | 0.973 | 370 |
|  | Systemic Lupus Erythematosus | 0.996 | 0.978 | 0.987 | 1.000 | 271 |
| **CatBoost** |  |  |  |  |  |  |
|  | Ankylosing Spondylitis | 0.783 | 0.518 | 0.623 | 0.969 | 425 |
|  | Normal | 0.863 | 0.844 | 0.854 | 0.979 | 321 |
|  | Psoriatic Arthritis | 0.830 | 0.902 | 0.864 | 0.968 | 357 |
|  | Reactive Arthritis | 0.508 | 0.932 | 0.658 | 0.960 | 103 |
|  | Rheumatoid Arthritis | 0.840 | 0.882 | 0.861 | 0.948 | 570 |
|  | Sjögren's Syndrome | 0.863 | 0.886 | 0.875 | 0.975 | 370 |
|  | Systemic Lupus Erythematosus | 0.996 | 0.978 | 0.987 | 1.000 | 271 |
