# Supplementary material for: Calibrated, explainable machine learning on routine laboratory data to characterize diagnostic assignment patterns in rheumatic diseases: a retrospective study of 12,085 patients
Source: BMC Rheumatol. 2025 Dec 29;10:10. doi: 10.1186/s41927-025-00607-7 (PMC12849087; doi:10.1186/s41927-025-00607-7)
Supplement: Supplementary file 9 — Supplementary Material 9 [file 41927_2025_607_MOESM9_ESM.docx]

**Supplementary Table S10: Seronegative vs Seropositive Biomarker Comparison**

| Biomarker | Seronegative (n=390) | Seropositive (n=11,695) | Difference | % Difference | p-value |
| --- | --- | --- | --- | --- | --- |
| ESR (mm/hr) | 24.4 ± 13.9 | 24.2 ± 14.2 | +0.21 | +0.9% | 0.756 |
| CRP (mg/L) | 13.7 ± 9.9 | 13.3 ± 10.1 | +0.39 | +2.9% | 0.412 |
| HLA-B27 | **0.703** | **0.660** | **+0.043** | **+6.5%** | **0.024** * |
| ANA | 0.621 | 0.652 | -0.031 | -4.8% | 0.134 |
| Anti-Ro | 0.649 | 0.607 | +0.042 | +6.9% | 0.065 |
| Anti-La | **0.554** | **0.627** | **-0.073** | **-11.6%** | **0.001** ** |
| Anti-dsDNA | 0.549 | 0.580 | -0.031 | -5.4% | 0.151 |
| Anti-Sm | 0.556 | 0.566 | -0.010 | -1.7% | 0.651 |
| C3 (mg/dL) | 135.2 ± 34.0 | 131.8 ± 34.3 | +3.43 | +2.6% | 0.048 * |
| C4 (mg/dL) | 38.4 ± 18.3 | 38.2 ± 18.6 | +0.26 | +0.7% | 0.773 |
