# Supplementary material for: Calibrated, explainable machine learning on routine laboratory data to characterize diagnostic assignment patterns in rheumatic diseases: a retrospective study of 12,085 patients
Source: BMC Rheumatol. 2025 Dec 29;10:10. doi: 10.1186/s41927-025-00607-7 (PMC12849087; doi:10.1186/s41927-025-00607-7)
Supplement: Supplementary file 10 — Supplementary Material 10 [file 41927_2025_607_MOESM10_ESM.docx]

**Supplementary Table S9: Confusion Matrix (Random Forest, n=2,417)**

|  | **Predicted →** | AS | Normal | PsA | Reactive | RA | Sjögren's | SLE |
| --- | --- | --- | --- | --- | --- | --- | --- | --- |
| **True ↓** |  |  |  |  |  |  |  |  |
| **AS** | 425 | **245** | 20 | 54 | 15 | 109 | 2 | 0 |
| **Normal** | 321 | 4 | **263** | 0 | 0 | 0 | 54 | 0 |
| **PsA** | 357 | 29 | 0 | **322** | 0 | 2 | 0 | 4 |
| **Reactive** | 103 | 15 | 0 | 0 | **83** | 5 | 0 | 0 |
| **RA** | 570 | 29 | 0 | 0 | 15 | **521** | 2 | 3 |
| **Sjögren's** | 370 | 0 | 31 | 0 | 0 | 2 | **337** | 0 |
| **SLE** | 271 | 0 | 3 | 0 | 0 | 0 | 3 | **265** |
