# Supplementary material for: Calibrated, explainable machine learning on routine laboratory data to characterize diagnostic assignment patterns in rheumatic diseases: a retrospective study of 12,085 patients
Source: BMC Rheumatol. 2025 Dec 29;10:10. doi: 10.1186/s41927-025-00607-7 (PMC12849087; doi:10.1186/s41927-025-00607-7)
Supplement: Supplementary file 11 — Supplementary Material 11 [file 41927_2025_607_MOESM11_ESM.docx]

**Supplementary Table S11: Seronegative Subtype Cluster Profiles (n=390)**

| **Feature** | **Subtype 1 (n=112)** | **Subtype 2 (n=98)** | **Subtype 3 (n=95)** | **Subtype 4 (n=85)** | **p-value** |
| --- | --- | --- | --- | --- | --- |
| **Cluster Characteristics** | HLA-B27+ High Inflammation | Anti-Ro+ Moderate Inflammation | Low Markers | ANA+ Low Complement |  |
|  |  |  |  |  |  |
| **Biomarkers (mean ± SD)** | | | | | |
| ESR (mm/hr) | 32.4 ± 12.8 | 24.6 ± 11.2 | 15.8 ± 8.9 | 26.1 ± 13.5 | <0.001 |
| CRP (mg/L) | 18.9 ± 10.4 | 14.2 ± 8.7 | 7.3 ± 6.2 | 13.8 ± 9.5 | <0.001 |
| **HLA-B27, n (%)** | **106 (94.6%)** | 52 (53.1%) | 48 (50.5%) | 58 (68.2%) | <0.001 |
| ANA, n (%) | 58 (51.8%) | 72 (73.5%) | 32 (33.7%) | **78 (91.8%)** | <0.001 |
| **Anti-Ro, n (%)** | 42 (37.5%) | **86 (87.8%)** | 38 (40.0%) | 64 (75.3%) | <0.001 |
| **Anti-La, n (%)** | 28 (25.0%) | **78 (79.6%)** | 22 (23.2%) | 48 (56.5%) | <0.001 |
| Anti-dsDNA, n (%) | 38 (33.9%) | 58 (59.2%) | 32 (33.7%) | 68 (80.0%) | <0.001 |
| Anti-Sm, n (%) | 42 (37.5%) | 54 (55.1%) | 38 (40.0%) | 64 (75.3%) | <0.001 |
| C3 (mg/dL) | 138.2 ± 28.4 | 136.8 ± 32.1 | 142.5 ± 29.8 | **118.4 ± 38.6** | <0.001 |
| C4 (mg/dL) | 39.8 ± 16.2 | 38.9 ± 17.8 | 41.2 ± 18.5 | **32.1 ± 19.4** | 0.002 |
|  |  |  |  |  |  |
| **Likely Disease** | | | | | |
| Primary diagnosis | Ankylosing Spondylitis | Sjögren's Syndrome | Seronegative RA or Normal | SLE or Undifferentiated CTD |  |
| Supporting features | High HLA-B27, High ESR/CRP | Anti-Ro/Anti-La positive | Minimal autoantibodies | ANA+, Low C3/C4 |  |
