# Supplementary figures and images for: Calibrated, explainable machine learning on routine laboratory data to characterize diagnostic assignment patterns in rheumatic diseases: a retrospective study of 12,085 patients
Source: BMC Rheumatol. 2025 Dec 29;10:10. doi: 10.1186/s41927-025-00607-7 (PMC12849087; doi:10.1186/s41927-025-00607-7)

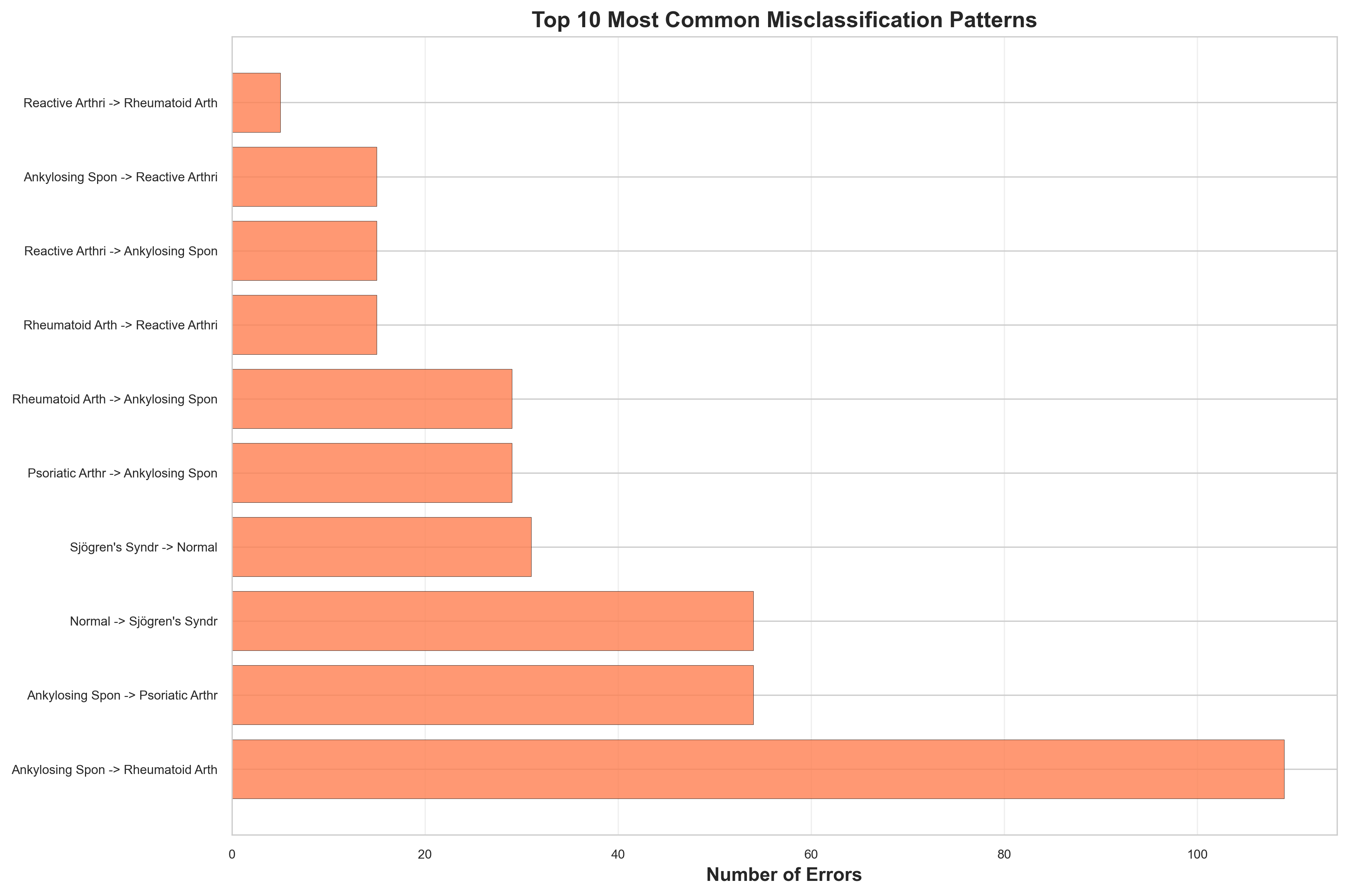

Supplement: Supplementary file 12 — Supplementary Material 12 [file 41927_2025_607_MOESM12_ESM.png]

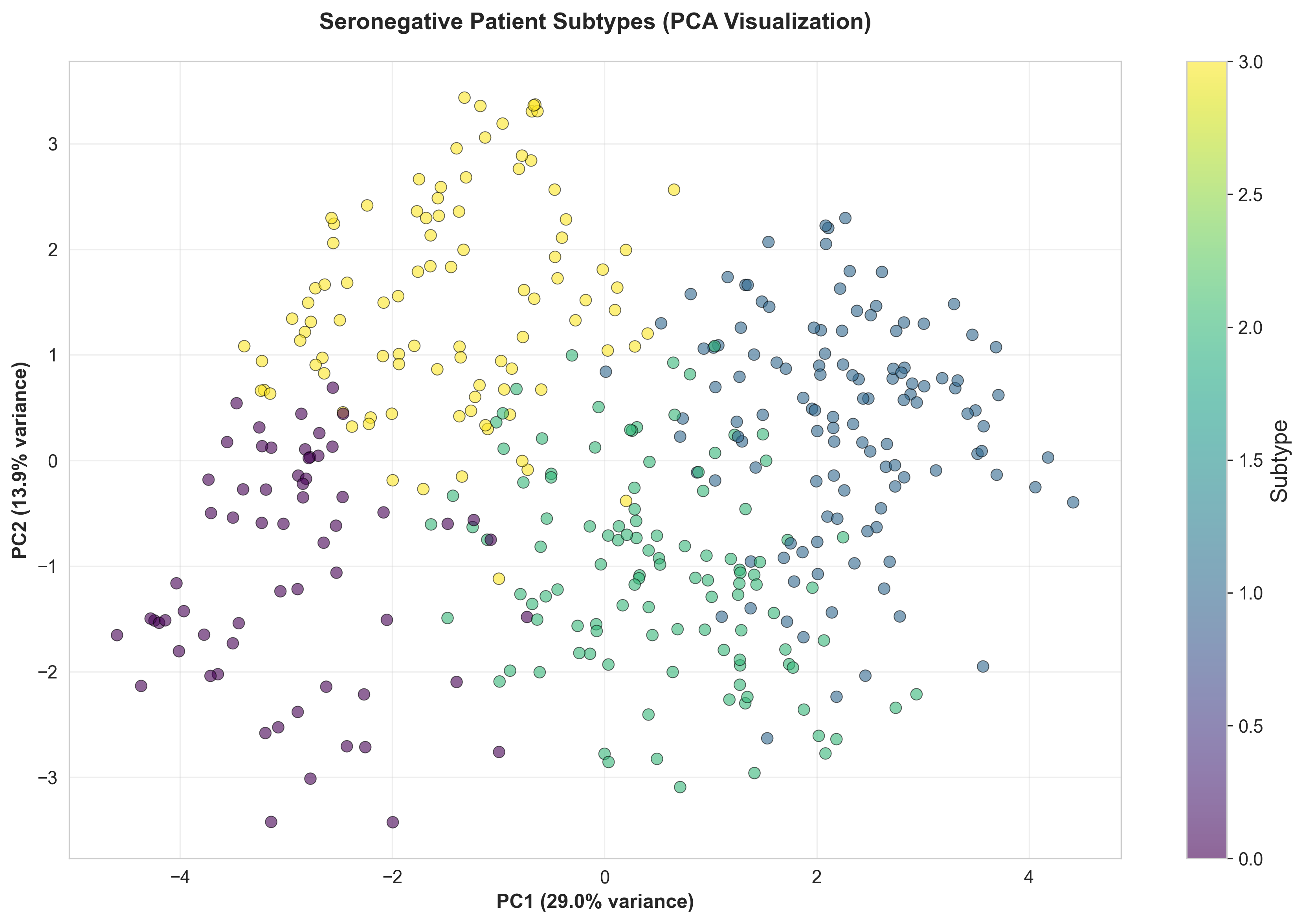

Supplement: Supplementary file 13 — Supplementary Material 13 [file 41927_2025_607_MOESM13_ESM.png]
